# Telengiectactic osteosarcoma

**DOI:** 10.4103/0973-029X.80011

**Published:** 2011

**Authors:** Jose Joy Idiculla, Renjith George, B Sivapathasundharam

**Affiliations:** *Department of Oral and Maxillofacial Pathology, Meenakshi Ammal Dental College, Maduravoyal, Chennai, India*

A 25-year-old man reported with swelling in the right lower jaw since five months. Clinically, a diffuse swelling Swelling on the posterior part of the mandible, right side measuring of mandible measuring 6 × 5 cm^2^ was noted. It was hard in consistency, with bicortical expansion. No scars or sinus discharge was present. Intraorally, obliteration of buccal vestibule and lingual cortical expansion were present. The swelling was tender, with no sign of paraesthesia. No intraoral discharge was present. Radiologically, the lesion had moth-eaten appearance, with mixed radiolucency.

## HISTOPATHOLOGY

- Numerous large irregular blood-filled channels (sinusoids) supported by connective tissue septae containing multinucleated giant cells and primitive-looking mesenchymal cells [Figures 1–6].
- Sinusoidal spaces partially lined by endothelial cells cells and containing extravasated RBCs.
- Highly pleomorphic and hyperchromatic mesenchymal cells resembling atypical osteoblasts were seen throughout the section [Figures 7–14].
- Osteoid formation and dystrophic calcifications were noted.

**Figures 1 to 4:**
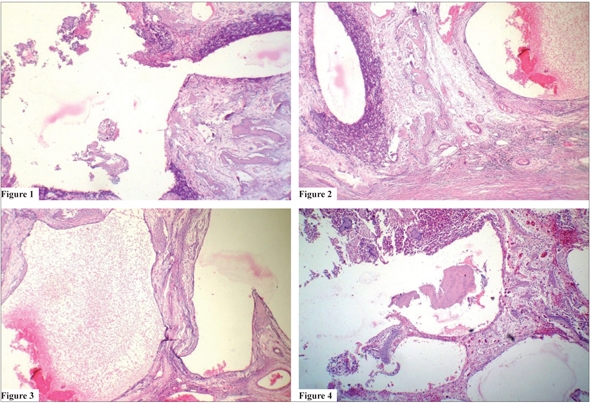
Sections showing numerous large endothelial lined spaces surrounded by tumor cells in certain areas. They almost resemble aneurysmal bone cyst under low power (H and E section, 4×)

**Figures 5 to 8:**
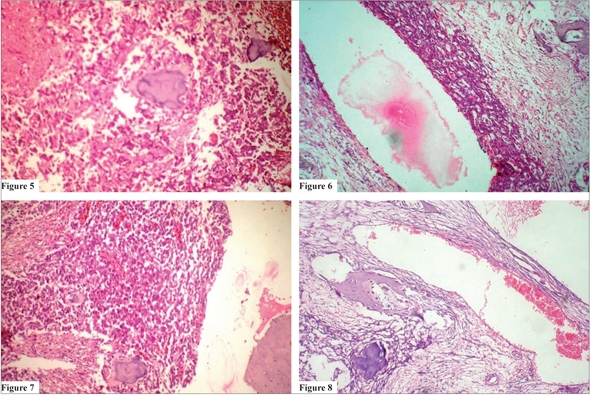
Sections showing tumor cells along with osteoid formation (H and E section, 10×)

**Figures 9 to 14:**
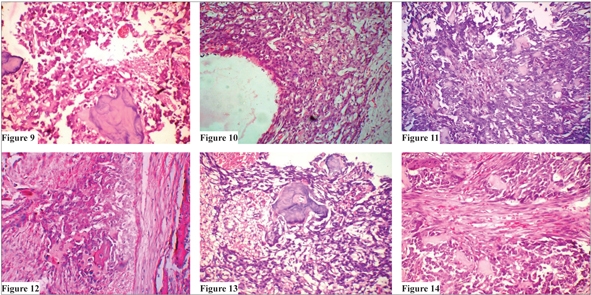
Sections showing malignant osteoblasts with osteoid production and dystrophic calcifications (H and E section, 40×)

## DIFFERENTIAL DIAGNOSIS

Aneurysmal bone cyst has thin rims with well-defined inner margins and septae surrounding the large blood-filled vascular spaces.

Conventional osteosarcoma shows tumor cells which are capable of producing extracellular matrix that may be osseous, cartilaginous or fibrous in various proportions with production of osteoid.

## FINAL DIAGNOSIS

Telangiectatic osteosarcoma

